# Evaluation of Chemical Composition and Antileishmanial and Antituberculosis Activities of Essential Oils of *Piper* Species

**DOI:** 10.3390/molecules21121698

**Published:** 2016-12-12

**Authors:** Karine Zanoli Bernuci, Camila Cristina Iwanaga, Carla Maria Mariano Fernandez-Andrade, Fabiana Brusco Lorenzetti, Eduardo Caio Torres-Santos, Viviane dos Santos Faiões, José Eduardo Gonçalves, Wanderlei do Amaral, Cícero Deschamps, Regiane Bertin de Lima Scodro, Rosilene Fressatti Cardoso, Vanessa Pietrowski Baldin, Diógenes Aparício Garcia Cortez

**Affiliations:** 1 Programa de Pós-Graduação em Ciências Farmacêuticas, Universidade Estadual de Maringá, Maringá 87020-900, PR, Brazil; kazanoli@hotmail.com (K.Z.B.); camila_iwanaga@hotmail.com (C.C.I.); carla.mfernandez@hotmail.com (C.M.M.F.-A.); fabi.bruschi@hotmail.com (F.B.L.); 2 Laboratório de Bioquímica de Tripanosomatídeos, Instituto Oswaldo Cruz, FIOCRUZ, Rio de Janeiro 21040-900, RJ, Brazil; ects@ioc.fiocruz.br (E.C.T.-S.); vsfaioes@ioc.fiocruz.br (V.d.S.F.); 3 Mestrado em Tecnologias Limpas e Mestrado em Promoção da Saúde, UniCesumar, Av. Guerdner, 1610, Jd. Aclimação, Maringá 87050-390, PR, Brazil; jose.goncalves@unicesumar.edu.br; 4 Instituto Cesumar de Ciências, Tecnologia e Inovação—ICETI, Av. Guerdner, 1610, Jd. Aclimação, Maringá 87050-390, PR, Brazil; 5 Setor de Ciências Agrárias/Departamento de Fitotecnia e Fitossanitaríssimo, Universidade Federal do Paraná, Curitiba 88035-050, PR, Brazil; institutobiointegral@gmail.com (W.d.A.); cicero@ufpr.br (C.D.); 6 Departamento de Análises Clínicas e Biomedicina, Universidade Estadual de Maringá, Maringá 87020-900, PR, Brazil; regianebertin@gmail.com (R.B.d.L.S.); rfressatticardoso@gmail.com (R.F.C.); vanessapbaldin@gmail.com (V.P.B.)

**Keywords:** Piperaceae, GC–MS, essential oil, *Leishmania amazonensis*, *Mycobacterium tuberculosis*

## Abstract

Essential oils from fresh Piperaceae leaves were obtained by hydrodistillation and analyzed by gas chromatography mass spectrometry (GC–MS), and a total of 68 components were identified. Principal components analysis results showed a chemical variability between species, with sesquiterpene compounds predominating in the majority of species analyzed. The composition of the essential oil of *Piper mosenii* was described for the first time. The cytotoxicity of the essential oils was evaluated in peritoneal macrophages and the oils of *P. rivinoides*, *P. arboretum*, and *P. aduncum* exhibited the highest values, with cytotoxic concentration at 50% (CC_50_) > 200 µg/mL. Both *P. diospyrifolium* and *P. aduncum* displayed activity against *Leishmania amazonensis*, and were more selective for the parasite than for the macrophages, with a selectivity index (SI) of 2.35 and >5.52, respectively. These SI values were greater than the 1 for the standard drug pentamidine. The antileishmanial activity of the essential oils of *P. diospyrifolium* and *P. aduncum* was described for the first time. *P. rivinoides, P. cernuum*, and *P. diospyrifolium* displayed moderate activity against the *Mycobacterium tuberculosis* H_37_Rv bacillus, with a minimum inhibitory concentration (MIC) of 125 µg/mL. These results are relevant and suggests their potential for therapeutic purposes. Nevertheless, further studies are required to explain the exact mechanism of action of these essential oils.

## 1. Introduction

The Piperaceae family comprises around 3600 species [1], which are distributed in tropical and subtropical regions in the northern and southern hemispheres. The family includes herbaceous plants, shrubs, and (less frequently) trees, and is distributed across eight genera. The *Piper* genus the most widely represented, with around 2000 species [2].

Species of *Piper* L. have previously been chemically investigated, resulting in the isolation of various substances with biological properties such as alkaloids, propenyl phenols, chalcones, dihydrochalcones, flavanones, flavones, amides, lignans, terpenes, and neolignans [3,4,5].

Biological properties of essential oils and extracts from plants of the genus *Piper*, including antibacterial [6,7,8], trypanocidal [9,10], antileishmanial [11,12], anti-inflammatory [13], antifungal [14,15], anti-*Mycobacterium* [16,17] and antioxidant [18,19,20] activities have been described. Considering these previously documented activities, the essential oil from *Piper* species shows potential for the development of new drugs for the treatment of neglected diseases such as leishmaniasis and tuberculosis, due to the resistance of the microorganisms involved. The major side effects of currently used drugs should also be considered.

Tuberculosis (TB) has been one of the most significant causes of suffering and death since the 19th century. The spread of the disease coincided with industrialization and the rapid and disorderly expansion of urban spaces. It is estimated that 9.6 million new TB cases occurred around the world in 2014. Of these, 5.4 million affected men, 3.2 million affected women, and 1.0 million cases involved children [21]. Leishmaniasis is among the most significant neglected diseases. World Health Organization data shows that it affects 350 million people in 88 countries, 72 of which are developing nations. Over the past decade, new endemic areas have emerged and the number of cases of the disease has increased [22].

The present study investigated the chemical composition and antileishmanial, cytotoxic, and anti-*Mycobacterium tuberculosis* activities of essential oils obtained from the leaves of *Piper* species.

## 2. Results and Discussion

### 2.1. Identification and Quantification of Essential Oil from Piper Species

A total of 68 components were identified from different species using GC–MS analysis. The compound identification percentage was over than 84% (Table 1). The composition (%) was obtained from the ratio between the integration of the total area of the chromatogram and the partial area of each peak. While chemical composition and essential oil content differed among species, the monoterpene and sesquiterpene compounds and (E)-caryophyllene were present in all the oils, with values ranging from 1.7% to 12.6%. The major compounds included α-thujene, α-pinene, β-pinene, limonene, β-phellandrene, safrole, δ-elemene, β-elemene, γ-elemene, α-humulene, dehydro-aromadendrene, trans-cadina-1(6), 4-diene, γ-gurjunene, bicyclogermacrene, (*Z*)-α-bisabolene, δ-cadinene, spathulenol, caryophyllene oxide, humulene epoxide II, epi-1-cubenol, epi-α-muurolol and α-muurolol. The essential oil composition of *P. mosenii* was described for the first time.

Table 2 shows the component percentages and the number of compounds. Of these, 15 were monoterpene and 53 were sesquiterpene in nature. The results show that the essential oil of this species was rich in sesquiterpene-type compounds. The essential oils of *P. xylosteoides* and *P. mikanianum*, unlike the other species, contained large amounts of monoterpene compounds.

The essential oil of the *Piper duckei* and *Piper demeraranum* leaves were also obtained by hydrodistillation (4 h) and analyzed by GC–MS. A total of 25 compounds were identified, and the results showed that these species are also rich in sesquiterpenes [24]. Analysis of the essential oil composition of the leaves of *Piper vicosanum*, with an extraction time of 4 h, identified the sesquiterpenes γ-elemene (14.16%) and α-alaskene (13.44%) and the monoterpene limonene (10.09%) as the majority substances [25]. The α-alaskene substance was not found in any of the nine species analyzed in the present study.

The difference in the chemical composition of the oils among the *Piper* species may be due to several factors, including genetic differences, circadian rhythms, seasonality, temperature, water availability, ultraviolet radiation, stage of development, time of collection, nutrients, soil characteristics, altitude, mechanical stimuli, and attack by herbivores or pathogens [26,27].

### 2.2. Principal Component Analysis (PCA)

**Figure 1:**
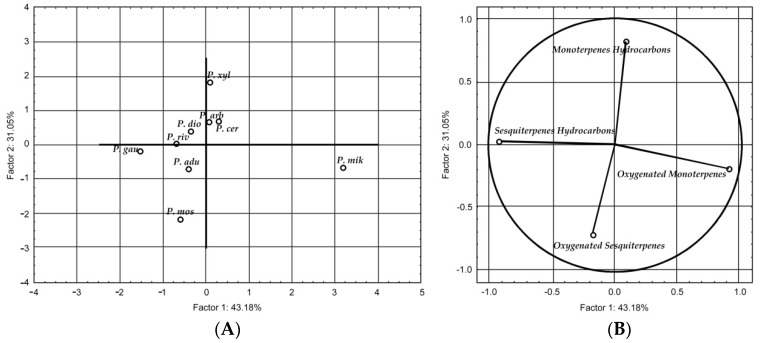
(**A**) Clustering of species by chemical groups; (**B**) Clustering of chemical constituents. For the abbreviations of the *Piper* species see Table 1.

The 68 compounds detected in the essential oils were subjected to PCA. Variance of 43.18% and 31.05% were detected in the horizontal and vertical axes, respectively, with a variance of approximately 74% among the components of the species (Figure 1).

The most chemically similar species *P. cernuum*, *P. aduncum*, *P. diospyrifolium*, *P. rivinoides*, and *P. gaudichaudianum*, with sesquiterpene hydrocarbon compounds the most prevalent. Unlike the other species, *P. xylosteoides* and *P. mikanianum* contained a large quantity of monoterpenes. *P. xylosteoides* was the richest in monoterpene hydrocarbons, while *P. mikanianum* was richest in oxygenated monoterpenes. *P. arboretum* and *P. mosenii* differed from the others as they had a greater composition of oxygenated sesquiterpene components. Various studies have demonstrated a preference of the *Piper* species to synthesize sesquiterpenes [28,29,30,31].

### 2.3. Leishmanicidal and Cytotoxicity Activities

Initially, the present study analyzed the cytotoxic activity of essential oils. Different CC_50_ (cytotoxic concentration at 50%) values were observed among species (Table 3), with the oils of *P. rivinoides*, *P. arboretum* and *P. aduncum* less toxic (CC_50_ values > 200 µg/mL). The essential oil of the aerial parts of *Piper auritum* had CC_50_ values of 106.4 ± 3.4 µg/mL against peritoneal macrophages from BALB/c mice [32]. *Piper hispidum* had a CC_50_ value of 35.5 µg/mL against peritoneal macrophages from BALB/c mice and CC_50_ > 100 against Vero cells [33]. The present study found higher values, which may be due to differences in the chemical composition of the oils and due to different assay conditions used.

The oils analyzed presented activity against promastigotes, with those of *P. rivinoides*, *P. mosenii, P. cernuum*, *P. diospyrifolium, P. arboretum*, and *P. aduncum* displaying the highest values, varying from 10.9 ± 2.7 µg/mL–27.1 ± 0.9 µg/mL. The oils of *P. diospyrifolium* and *P. aduncum* inhibited the growth of axenic amastigote forms, with IC_50_ (inhibitory concentration at 50%) values of 76.1 ± 9.0 µg/mL and 36.2 ± 2.9 µg/mL, respectively. Both these essential oils were more selective for the parasite than for macrophages, with a selectivity index (SI) of 2.35 for *Piper diospyrifolium* and SI > 5.52 for *Piper aduncum.* These SI values were greater than the one for the standard drug pentamidine. Here, the leishmanicidal activity of the essential oils of *Piper diospyrifolium* and *Piper aduncum* is described for the first time.

Studies carried out with the essential oil of *Piper hispidum* identified an IC_50_ of 3.4 µg/mL against amastigote forms of *Leishmania amazonensis* [33]. The leishmanicidal activity of the essential oils is due to the presence of terpenoids, which have demonstrated antiparasitic activity against a range of species of *Leishmania* [34].

The leishmanicidal action of a number *Piper* species has been evaluated, with the essential oils of the leaves of *Piper demeraranum* and *Piper duckei* presenting an IC_50_ of 86.0 ± 2.4 µg/mL and 46.0 ± 1.3 µg/mL, respectively, against promastigote forms of *Leishmania amazonensis* [24]. The action of the essential oil of *Piper cubeba* against promastigote forms of *Leishmania amazonensis* were evaluated, although the oil was not active at the various concentrations tested. The authors suggested this was because the oil exhibited mainly monoterpene compounds, which corresponded to 90% of the oil [35]. Therefore, we can consider that the presence of more sesquiterpene compounds contributes to leishmanicidal activity. In the present study, the most active essential oils comprised a large number of sesquiterpenes, while the essential oils of *P. xylosteoides* and *P. mikanianum*, which contained more monoterpene compounds, did not demonstrate leishmanicidal activity.

These results are relevant and promising for in vitro tests. Nevertheless, further studies are required to explain the mechanism of action.

### 2.4. Anti-M. tuberculosis Activity

Natural products and their derivatives have been found to display inhibitory activity against the growth of *M. tuberculosis*, while some have been selected as prototype molecules for the development of new antituberculosis agents [36,37].

Evaluation of the anti-*M. tuberculosis* activity of the essential oils of Piperaceae identified minimum inhibitory concentration (MIC) values equal to or greater than 125 µg/mL (Table 4). The essential oils of *Piper rivinoides*, *Piper cernuum*, and *Piper diospyrifolium* can therefore be considered to possess moderate activity. MIC values <100 µg/mL are ideal candidates against *M. tuberculosis*, while values of 100–200 µg/mL are considered moderate candidates [38].

After determining the MIC of the oils, the Selectivity index (SI) was calculated. Only the essential oil of *Piper rivinoides* had an SI greater than 1, with a value of 1.6. To increase this value, the main components of the oil can be fractionated and/or isolated, as it has been established that the high lipophilicity of terpenes, which are rich in mycolic acid (lipophilic), is probably the main factor in their penetration of the cell wall of the mycobacteria [39].

Previous evaluation of the antimycobacterial action of the essential oils of *P. auritum* and *P. bogotense* obtained MIC values of 400 ± 220 and 130 ± 95 µg/mL [38], respectively. These results were superior to those of the present study. Research into essential oils is important for the treatment of tuberculosis, as this pathogen preferably settles in the lungs, where it remains active and can trigger the symptoms of the disease. Some studies have already initiated the use of essential oils in an inhalation form in anti-TB treatment [40]; following inhalation, the essential oil moves into the bronchi and then reaches the alveoli of the patient, spreading into the pulmonary capillaries, where it can exert local and systemic effects.

## 3. Materials and Methods

### 3.1. Plant Materials

The specimens of *Piper rivinoides* Kunth, *Piper mosenii* C. DC., *Piper cernuum* Vell., *Piper diospyrifolium* Kunth, *Piper arboretum* Aubl., *Piper aduncum* L., *Piper gaudichaudianum* Kunth, *Piper xylosteoides* (Kunth) Steud. and *Piper mikanianum* (Kunth) Steudelwere collected between April and October 2014 in Antonina and Cerro Azul, in the state of Paraná, and Atalanta, in the state of Santa Catarina, Brazil (Table 5). The plants were identified by the botanist José Tadeu Weidlich Motta, and a voucher specimen was deposited at the Herbarium of the Municipal Botanical Museum Curitiba. The essential oil was extracted from fresh leaves.

### 3.2. Extraction of Essential Oil

Essential oils were obtained from fresh leaves (600 g) by hydrodistillation in a Clevenger apparatus for 4 h with 600 mL of water. At the end of each distillation, the oils were collected, centrifuged at 5000 rpm for 2 min, transferred to glass, and stored at a temperature of −4 °C.

### 3.3. GC–MS Analysis

The analysis of the essential oil was carried out in a gas chromatograph (Agilent 7890 B, Agilent Technologies, Santa Clara, CA, USA) coupled to a mass spectrum (Agilent 5977 A) equipped with an Agilent HP-5 MS UI capillary column (30 m × 0.250 mm × 0.25 µm). To carry out the analysis, the essential oils were diluted to 5% in dichloromethane and injected under the following conditions: injector temperature of 220 °C, injection volume 1 µL at a ratio of 1:20 (split mode), initial column temperature of 60 °C heated gradually to 180 °C at a 2 °C/min rate, heated to 220 °C at a 10 °C/min rate, and then to 300 °C at 40 °C/min. The carrier gas (helium) flow was set at 1 mL·min^−1^. The temperatures of the transfer line, ion source, and quadrupole were 250, 230, and 150 °C, respectively. The mass spectra were obtained at a range of 40–450 (*m/z*) in scan mode with a solvent delay time of 3 min. The compounds were identified based on comparison of their retention indices (RI) obtained using various *n*-alkanes (C7–C30). The electron ionization (EI)-mass spectra were compared with Wiley library spectra and according to Adams [23].

### 3.4. Principal Components Analysis (PCA)

Clustering analysis of species was performed with the unweighted pair-group method using arithmetic average (UPGMA) algorithm based on squared Euclidean distances. Prior to the calculation of these distances, the data was standardized to obtain a mean of zero and a variance of one. Principal components analysis was then applied using the primary data as the covariance matrix [41,42]. These analyses were performed using the Statistica software package, version 12.0 (StatSoft, Tulsa, OK, USA).

### 3.5. Cytotoxicity

BALB/c mice macrophages were obtained by peritoneal lavage with a cold RPMI medium (Sigma-Aldrich, St. Louis, MO, USA). The macrophages were placed in RPMI culture medium (pH 7.2, supplemented with 10% fetal bovine serum) in 96-well plates at a ratio of 2 × 10^6^ cells/well and incubated with essential oils (0–250 μg/mL) for 72 h at 37 °C under 5% CO_2_. After removing the supernatant, viable cells were quantified by adding 22 µL of resazurin solution per well (500 µM) in phosphate-buffered saline (PBS). Fluorescence was measured using a Spectra Max M2 spectrofluorometer (Molecular Devices, Silicon Valley, CA, USA) under excitation and at emission wavelengths of 560 nm and 590 nm, respectively. The percentage of viable cells relative to the control cells was calculated. The tests were carried out in triplicate. The concentration effect curves were fitted with nonlinear regression using Graph Pad Prism 5.0 (Graph Pad Software, San Diego, CA, USA), and the CC_50_ values were determined.

### 3.6. Antipromastigote Activity

Promastigotes of *L. amazonensis* (MHOM/BR/77/LTB0016) were maintained in flasks at 26 °C in Schneider’s medium (Sigma-Aldrich Corp., St. Louis, MO, USA) supplemented with 10% fetal bovine serum. Tests were performed in 96-well plates with an initial inoculum of 1.0 × 10^6^ parasites/mL incubated with essential oils (0–200 μg/mL) for 72 h at 26 °C. After incubation, antileishmanial activity was evaluated by adding 22 µL of resazurin solution per well (500 µM, Sigma-Aldrich). After 4 h, fluorescence was measured using a Spectra Max M2 spectrofluorometer (Molecular Devices) under excitation and at emission wavelengths of 560 nm and 590 nm, respectively. The tests were carried out in triplicate. The concentration effect curves were fitted using nonlinear regression with Graph Pad Prism 5.0, and the IC_50_ values were determined.

### 3.7. Axenic Amastigotes

*L. amazonensis* axenic amastigotes were obtained as previously described [43]. Briefly, stationary phase *L. amazonensis* promastigotes were washed in cold PBS and incubated in Schneider’s medium (Sigma-Aldrich), pH 5.5, supplemented with 20% fetal bovine serum and maintained at 32 °C for 5 days to induce differentiation. Subcultures were obtained at one-week intervals under the same conditions. For antiamastigote assays, axenic amastigotes (5.0 × 10^6^/mL) were incubated with essential oils (0–200 μg/mL) for 72 h. After incubation, activity was evaluated by adding 22 µL of resazurin solution (500 µM) to each well. After 4 h, fluorescence was measured using a Spectra Max M2 spectrofluorometer (Molecular Devices) under excitation and at emission wavelengths of 560 nm and 590 nm, respectively. The tests were carried out in triplicate. The concentration effect curves were fitted using nonlinear regression with Graph Pad Prism 5.0, and the IC_50_ values were determined.

### 3.8. Anti-Mycobacterium tuberculosis Activity Assay

The anti-*M. tuberculosis* activities of essential oils from leaves of *Piper* species as evaluated by colorimetric resazurin microtiter assay (REMA) plate method [44]. Briefly, 200 μL of sterile distilled water was distributed in the outer wells of the microplate (Falcon 3072, Becton Dickinson, Lincoln Park, NJ, USA); the essential oils were diluted in dimethylsulfoxide (DMSO, Amresco, Solon, OH, USA) and serial twofold dilutions from 250 to 1.9 μg/mL were carried out in Middlebrook 7H9 broth (Difco Laboratories, Detroit, MI, USA) supplemented with oleic acid, bovine albumin, dextrose, and catalase (OADC) Enrichment (BBL/Becton-Dickinson, Sparks, MD, USA). Isoniazid (Difco Laboratories, Detroit, MI, USA) was used as the reference drug at concentrations ranging from 0.007 to 1.0 μg/mL. One hundred microliters of each bacterial inoculum (*M. tuberculosis* H_37_Rv (ATCC 27294)), standardized at 1 McFarland turbidity and diluted to 1:20 in OADC-supplemented Middlebrook 7H9 broth, was inoculated into the wells. The plates were covered with lids and their edges were sealed with polyethylene tape. The plates were placed in a plastic box and incubated in a normal atmosphere for 7 days at 35 °C. The MIC readings were carried out after the addition of 30 μL of freshly prepared 0.01% resazurin solution (Acros, Morris Plains, NJ, USA) to each well, and the plates were incubated for 24–48 h at 35 °C. A color change from blue to pink indicated mycobacterial growth, and the MIC was the lowest extract concentration that prevented the color change. Medium, drug sterility, and bacterial growth with and without 2.5% (*v*/*v*) DMSO controls were included in all tests. The tests were carried out in triplicate.

## 4. Conclusions

The essential oils obtained from nine *Piper* species are composed of monoterpenes and sesquiterpenes, and the differences in their composition can be attributed to genetic differences and climatic and soil factors. The chemical composition of the essential oil of *Piper mosenii* is described here for the first time. The essential oils of *Piper diospyrifolium* and *Piper aduncum* were most active against *L. amazonensis* and the oil of *Piper rivinoides*, *Piper cernuum*, and *Piper diospyrifolium* were most active against *Mycobacterium tuberculosis.* These results are relevant and suggest their potential for therapeutic purposes. Nevertheless, further studies are required to explain the exact mechanism of action these essential oils.

## Figures and Tables

**Table 1:** Chemical composition of essential oil from *Piper* species.

Peak	Compounds	RIa	*P. riv*		*P. mos*		*P. cer*		*P. dio*		*P. arb*		*P. adu*		*P. gau*		*P. xyl*		*P. mik*	
			RI	%	RI	%	RI	%	RI	%	RI	%	RI	%	RI	%	RI	%	RI	%
***Monoterpene Hydrocarbons***																				
**1**	α-Thujene	930	-	-	931	1.9	-	-	-	-	-	-	-	-	-	-	934	7.9	934	6.0
**2**	α-Pinene	**939**	932	4.4	-	-	933	11.4	933	6.7	-	-	-	-	-	-	-	-	946	1.1
**3**	β-Pinene	**983**	976	3.7	976	3.8	976	7.9	975	1.2	-	-	-	-	-	-	-	-	-	-
**4**	Myrcene	**990**	-	-	-	-	-	-	-	-	-	-	-	-	-	-	992	2.8	-	-
**5**	α-Phellandrene	**1002**	1005	1.1	-	-	-	-	-	-	-	-	-	-	-	-	1006	3.8	-	-
**6**	δ-3-Carene	**1011**	-	-	-	-	-	-	-	-	-	-	-	-	1012	5.9	-	-	-	-
**7**	ρ-Cymene	**1024**	-	-	1023	1.5	-	-	-	-	-	-	-	-	1026	1.2	-	-	-	-
**8**	o-Cymene	1026	-	-	-	-	1022	1.2	-	-	-	-	-	-	-	-	-	-	-	-
**9**	Limonene	1029	-	-	-	-	-	-	1029	6.7	-	-	-	-	-	-	-	-	1027	1.8
**10**	β-Phellandrene	1029	-	-	-	-	-	-	-	-	-	-	-	-	-	-	1033	22.6	-	-
**11**	Sylvestrene	**1030**	1026	1.2	-	-	-	-	-	-	-	-	-	-	-	-	-	-	-	-
**12**	(*Z*)-β-Ocimene	**1037**	-	-	-	-	-	-	1036	1.5	-	-	1038	7.0	-	-	-	-	-	-
**13**	(*E*)-β-Ocimene	**1050**	-	-	-	-	-	-	-	-	-	-	1051	13.9	-	-	-	-	-	-
***Oxygenated Monoterpenes***																				
**14**	Linalool	**1096**	-	-	-	-	-	-	-	-	1100	1.7	1101	1.3	-	-	1101	1.2	-	-
**15**	Safrole	**1287**	-	-	-	-	-	-	-	-	-	-	1288	6.2	-	-	-	-	1309	72.4
***Sesquiterpene Hydrocarbons***																				
**16**	δ-EIemene	**1338**	-	-	-	-	-	-	-	-	1335	5.6	-	-	-	-	1337	6.6	-	-
**17**	α-Copaene	**1376**	-	-	-	-	-	-	1373	5.4	-	-	-	-	-	-	-	-	-	-
**18**	β-Elemene	**1390**	1388	1.6	-	-	1391	10.1	1390	3.0	1388	2.1	-	-	1390	3.5	1389	1.6	-	-
**19**	α-Gurjunene	**1409**	-	-	1404	1.3	-	-	-	-	-	-	-	-	-	-	-	-	-	-
**20**	(*E*)-Caryophyllene	**1419**	1415	6.6	1417	8.6	1415	6.9	1416	7.4	1415	12.6	1414	2.6	1414	1.7	1416	7.0	1416	2.4
**21**	β-Gurjunene	**1433**	-	-	-	-	-	-	-	-	-	-	1433	2.3	-	-	-	-	-	-
**22**	γ-Elemene	**1436**	-	-	-	-	-	-	-	-	-	-	-	-	1432	5.4	-	-	-	-
**23**	α-Guaiene	**1439**	-	-	1433	1.8	-	-	1435	2.5	-	-	-	-	-	-	-	-	-	-
**24**	α-Humulene	**1452**	1457	10.0	1452	11.3	1446	1.0	1448	1.6	1447	3.7	1449	4.9	1448	2.2	-	-	-	-
**25**	allo-Aromadendrene	**1460**	-	-	1456	2.1	-	-	-	-	-	-	1455	1.1	1456	2.3	-	-	-	-
**26**	Dehydro-aromadendrene	**1462**	1477	7.8	-	-	-	-	-	-	-	-	-	-	-	-	-	-	-	-
**27**	trans-Cadina-1(6),4-diene	**1476**	-	-	1473	1.7	-	-	-	-	1476	9.6	1475	1.1	-	-	-	-	-	-
**28**	γ-Gurjunene	**1477**	-	-	-	-	-	-	1478	6.9	-	-	-	-	1476	2.9	1477	4.7	-	-
**29**	γ-Muurolene	**1479**	-	-	1476	1.4	-	-	-	-	-	-	-	-	-	-	-	-	-	-
**30**	γ-Himachalene	**1482**	-	-	1481	1.5	-	-	1481	1.5	-	-	-	-	-	-	-	-	-	-
**31**	Germacrene D	1485	-	-	-	-	-	-	-	-	-	-	-	-	1489	1.7	-	-	1486	1.2
**32**	Aristolochene	1488	1481	1.8	-	-	-	-	-	-	-	-	-	-	-	-	-	-	-	-
**33**	β-Selinene	1490	-	-	-	-	-	-	1489	2.0	-	-	-	-	-	-	-	-	-	-
**34**	δ-Selinene	1492	-	-	-	-	1489	4.1	-	-	-	-	-	-	-	-	-	-	-	-
**35**	epi-Cubebol	1494	-	-	-	-	-	-	-	-	1490	4.5	-	-	-	-	-	-	-	-
**36**	α-Selinene	**1498**	-	-	-	-	-	-	1496	3.4	-	-	-	-	-	-	-	-	-	-
**37**	Bicyclogermacrene	**1500**	1495	11.8	1493	7.4	-	-	1501	2.3	-	-	1497	20.9	1493	4.4	1494	7.2	1493	3.1
**38**	α-Muurolene	**1500**	-	-	-	-	1497	1.7	-	-	-	-	-	-	-	-	-	-	-	-
**39**	(*Z*)-α-Bisabolene	**1507**	1506	10.9	-	-	-	-	-	-	-	-	-	-	1497	2.5	-	-	-	-
**40**	Germacrene A	**1509**	-	-	-	-	1499	3.4	1503	2.0	-	-	-	-	1501	2.6	-	-	-	-
**41**	α-Bulnesene	**1509**	-	-	-	-	-	-	-	-	-	-	-	-	-	-	-	-	1510	1.4
**42**	γ-Cadinene	**1513**	-	-	1510	2.1	-	-	-	-	1510	2.6	1514	5.5	-	-	-	-	-	-
**43**	Cubebol	**1515**	-	-	-	-	1510	2.0	-	-	-	-	-	-	1510	1.5	-	-	-	-
**44**	trans-Calamenene	**1522**	-	-	1520	2.3	-	-	-	-	-	-	-	-	-	-	-	-	-	-
**45**	β-Sesquiphellandrene	**1522**	1521	4.2	-	-	-	-	-	-	-	-	-	-	-	-	1516	1.1	-	-
**46**	δ-Cadinene	**1523**	-	-	-	-	-	-	1520	1.8	1520	2.0	1522	3.8	1565	45.3	1520	2.1	-	-
**47**	Germacrene B	**1561**	-	-	-	-	-	-	1553	6.7	1549	2.5	-	-	-	-	1550	1.4	-	-
**48**	(*E*)-Nerolidol	**1563**	-	-	-	-	1566	1.8	-	-	1565	1.5	-	-	-	-	1568	8.5	1565	1.9
***Oxygenated Sesquiterpenes***																				
**49**	Palustrol	**1568**	-	-	1560	1.3	-	-	-	-	-	-	-	-	-	-	-	-	-	-
**50**	Spathulenol	**1578**	1574	5.1	-	-	1575	11.5	-	-	1574	7.9	1575	5.3	1575	1.4	-	-	-	-
**51**	trans-Sesquisabinene hydrate	**1579**	1578	3.5	-	-	-	-	-	-	-	-	-	-	-	-	-	-	-	-
**52**	Caryophyllene oxide	**1583**	-	-	1579	12.1	1578	5.1	1576	2.5	1577	5.9	-	-	-	-	-	-	-	-
**53**	Globulol	**1590**	-	-	1581	4.8	-	-	-	-	-	-	-	-	-	-	-	-	-	-
**54**	Viridiflorol	**1592**	-	-	1590	5.8	1594	1.1	1586	2.6	-	-	-	-	-	-	-	-	-	-
**55**	Rosifoliol	**1600**	-	-	-	-	-	-	-	-	-	-	1596	1.4	-	-	-	-	-	-
**56**	Ledol	**1602**	1598	3.6	1599	3.1	-	-	-	-	-	-	-	-	-	-	-	-	-	-
**57**	Humulene epoxide II	**1608**	-	-	1607	6.3	-	-	-	-	1601	1.5	1603	1.6	-	-	-	-	-	-
**58**	10-epi-γ-Eudesmol	**1623**	-	-	-	-	-	-	-	-	1611	1.6	-	-	-	-	-	-	-	-
**59**	1-epi-Cubenol	**1628**	-	-	-	-	-	-	1624	3.1	1628	10.4	1623	1.3	-	-	1626	2.7	-	-
**60**	α-Acorenol	**1633**	1629	1.1	1628	1.5	-	-	-	-	-	-	-	-	-	-	-	-	-	-
**61**	epi-α-Cadinol	**1640**	1637	2.2	-	-	-	-	1637	2.6	1631	1.7	1637	3.5	1637	1.6	-	-	-	-
**62**	allo-Aromadendrene epoxide	**1641**	-	-	-	-	-	-	-	-	1634	1.5	-	-	-	-	-	-	-	-
**63**	epi-α-Muurolol	**1642**	-	-	-	-	1639	6.2	-	-	1638	3.6	-	-	-	-	1637	1.7	-	-
**64**	α-Muurolol	**1646**	1643	1.2			1643	5.8	-	-	-	-	1642	1.2	-	-	-	-	-	-
**65**	α-Cadinol	**1654**	1651	3.1	1651	2.8	1650	4.1	-	-	1651	5.4	1651	2.0	1646	3.3	1650	2.0	-	-
**66**	Selin-11-en-4-α-ol	**1659**	-	-	-	-	-	-	1656	17.7	1663	1.5	-	-	1662	1.2	-	-	-	-
**67**	Shyobunol	**1689**	-	-	-	-	-	-	-	-	-	-	1684	2.0	1683	1.2	-	-	-	-
**68**	Longifolol	**1714**	-	-	-	-	1714	1.2	-	-	-	-	-	-	1713	1.2	-	-	-	-
	**Total identified**			**85.2**		**86.4**		**86.5**		**91.1**		**89.4**		**88.9**		**93.0**		**84.9**		**91.3**

Methods of Identification: RI—Retention index calculated using C7–C30 *n*-alkane standard solution in an HP-5 MS UI Agilent (30 m × 0.250 mm × 0.25 µm) column. RIa—Relative retention index found in literature in capillary column HP-5 and comparison of the retention indices and/or mass spectra from literature [23]. -: Not detected. Identification based on comparison with Wiley library mass spectra. %—values of areas. Compounds listed in order of elution by column. *P. riv: Piper rivinoides* Kunth; *P. mos: Piper mosenii* C. DC.; *P. cer: Piper cernuum* Vell.; *P. dio: Piper diospyrifolium* Kunth; *P. arb: Piper arboretum* Aubl.; *P. adu: Piper aduncum* L.; *P. gau: Piper gaudichaudianum* Kunth; *P. xyl: Piper xylosteoides* (Kunth) Steud.; *P. mik: Piper mikanianum* (Kunt).

**Table 2:** Percentage of constituents per species group and number of compounds.

Group	*Monoterpenes Hydrocarbons*	*Oxygenated Monoterpenes*	*Sesquiterpene Hydrocarbons*	*Oxygenated Sesquiterpenes*
No. Compounds	13	2	33	20
*P. riv*	10.4	0	54.7	20.1
*P. mos*	7.2	0	41.5	37.7
*P. cer*	20.5	0	31.0	35.0
*P. dio*	16.1	0	46.5	28.5
*P. arb*	0	1.7	46.7	41.0
*P. adu*	20.9	7.5	42.2	18.3
*P. gau*	7.1	0	76.0	9.9
*P. xyl*	37.1	1.2	40.2	6.4
*P. mik*	8.9	72.4	10.0	0

For the abbreviation for the *Piper* species see Table 1.

**Table 3:** Cytotoxicity and leishmanicidal activities of essential oils from *Piper* species.

Essential Oils	*L. amazonensis* IC_50_/µg/mL		BALB/c Mice Macrophages CC_50_/µg/mL	SI = CC_50_/IC_50_ Axenic Amastigotes
	Promastigotes	Axenic Amastigotes		
*P. riv*	10.9 ± 2.7	>200	>200	-
*P. mos*	17.4 ± 5.0	>200	117 ± 3.0	-
*P. cer*	27.1 ± 0.9	>200	118.6 ± 5.4	-
*P. dio*	13.5 ± 0.4	76.1 ± 9.0	179.1 ± 1.0	2.35
*P. arb*	15.2 ± 2.4	>200	>200	-
*P. adu*	25.9 ± 1.3	36.2 ± 2.9	>200	>5.52
*P. gau*	93.5 ± 1.6	-	87.3 ± 0.04	-
*P. xyl*	>100	-	>100	-
*P. mik*	>100	-	>100	-
Pentamidine	2.84 ± 0.09	4.3 ± 1.2	5.03 ± 1.25	1.16

Values represent the mean ± standard deviation of three independent experiments. CC_50_: cytotoxic concentration at 50%; IC_50_: inhibitory concentration at 50%; SI: selectivity index (CC_50_/IC_50_ in axenic amastigotes). For abbreviations for *Piper* species see Table 1.

**Table 4:** Anti-*M. tuberculosis* H_37_Rv activity and cytotoxicity of Piperaceae essential oils.

Essential Oils	*M. tuberculosis* H_37_Rv MIC (µg/mL)	BALB/c Mice Macrophages CC_50_/µg/mL	SI = CC_50_/MIC
*P. riv*	125	> 200	>1.6
*P. mos*	250	117 ± 3.0	-
*P. cer*	125	118.6 ± 5.4	-
*P. dio*	125	179.1 ± 1.0	-
*P. arb*	>250	>200	-
*P. adu*	>250	>200	-
*P. gau*	>250	>100	-
*P. xyl*	>250	>100	-
*P. mik*	>250	87.3 ± 0.04	-
Isoniazid	0.06	NA	-

Values represent the mean ± standard deviation of three independent experiments. MIC: minimum inhibitory concentration; CC_50_: cytotoxic concentration in 50% off cells; SI: selectivity index (CC_50_/MIC). For the abbreviations of the Piper species see Table 1. NA: not analyzed.

**Table 5:** General data of native species of Piperaceae family collected for extraction in the municipalities of Antonina and Cerro Azul in Parana and Atalanta in Santa Catarina, 2014.

Scientific Name	No. Herbarium *	Municipality	Localization **			Collection Date
			Latitude	Longitude	Altitude (m)	
*Piper rivinoides* Kunth	396414	Antonina, PR	S 25°29.693′	W 49°00.844′	000	2 April 2014
*Piper mosenii* C. DC.	396409	Antonina, PR	S 25°29.693′	W 49°00.844′	000	2 April 2014
*Piper cernuum* Vell.	396416	Antonina, PR	S 25°29.693′	W 49°00.844′	000	2 April 2014
*Piper diospyrifolium* Kunth	396413	Antonina, PR	S 25°29.693′	W 49°00.844′	000	2 April 2014
*Piper arboretum* Aubl.	396412	Antonina, PR	S 25°29.693′	W 49°00.844′	000	2 April 2014
*Piper aduncum* L.	396411	Cerro Azul, PR	S 24°45.863′	W 49°16.368′	528	5 April 2014
*Piper gaudichaudianum* Kunth	396403	Antonina, PR	S 25°29.693′	W 49°00.844′	000	24 September 2014
*Piper xylosteoides* (Kunth) Steud.	396405	Cerro Azul, PR	S 24°45.863′	W 49°16.368′	528	1 October 14
*Piper mikanianum* (Kunth) Steudel	396408	Atalanta, SC	S 25°29.830′	W 49°00.919′	640	8 October 14

* Specimen number referring to voucher specimen identified, as found in the MBM Herbarium in Curitiba, Parana. ** Coordinates of species collection, with an average error of 15 m around the collection point.
